# The effects of vegetable pickling conditions on the dynamics of microbiota and metabolites

**DOI:** 10.7717/peerj.11123

**Published:** 2021-04-06

**Authors:** Kazunori Sawada, Hitoshi Koyano, Nozomi Yamamoto, Takuji Yamada

**Affiliations:** 1 Innovation Division, Gurunavi, Inc., Chiyoda-ku, Tokyo, Japan; 2 School of Life Science and Technology, Tokyo Institute of Technology, Meguro-ku, Tokyo, Japan

**Keywords:** Pickle production, Halophilic bacteria, Branched-chain amino acids, Salting process, Fermented pickle

## Abstract

**Background:**

Salting is a traditional procedure for producing pickled vegetables. Salting can be used as a pretreatment, for safe lactic acid fermentation and for salt stock preparation. This study aimed to provide valuable knowledge to improve pickle production by investigating the dynamics of microbiota and metabolites during the pretreatment and salt stock preparation processes, which have previously been overlooked. The differences in these process conditions would be expected to change the microbiota and consequently influence the content of metabolites in pickles.

**Methods:**

Samples, collected from eight commercial pickle manufacturers in Japan, consisted of the initial raw materials, pickled vegetables and used brine. The microbiota were analyzed by 16S rRNA sequencing and the metabolites quantified by liquid chromatograph-mass spectrometry. Statistical analyses helped to identify any significant differences between samples from the initial raw materials, pretreatment process and salt stock preparation process groups.

**Results:**

Under pretreatment conditions, aerobic and facultative anaerobic bacteria were predominant, including *Vibrio*, a potentially undesirable genus for pickle production. Under salt stock preparation conditions, the presence of halophilic bacteria, *Halanaerobium*, suggested their involvement in the increase in pyruvate derivatives such as branched-chain amino acids (BCAA). PICRUSt analysis indicated that the enhanced production of BCAA in salt stock was caused not by quantitative but by qualitative differences in the biosynthetic pathway of BCAA in the microbiota.

**Conclusion:**

The differences in the microbiota between pretreatment and previously studied lactic acid fermentation processes emphasized the importance of anaerobic conditions and low pH under moderate salinity conditions for assuring safe pickle production. The results from the salt stock preparation process suggested that the *Halanaerobium* present may provide a key enzyme in the BCAA biosynthetic pathway which prefers NADH as a coenzyme. This feature can enhance BCAA production under anaerobic conditions where NADH is in excess. The effects shown in this study will be important for adjusting pickling conditions by changing the abundance of bacteria to improve the quality of pickled vegetables.

## Introduction

Pickled vegetables are produced using knowledge accrued since ancient times (Bautista-Gallego et al., 2013; Chakraborty & Roy, 2018). Although the modern food industry makes pickles using refrigeration technologies and chemical additives, they are still made with salt (sodium chloride) and vinegar (acetate) (Lupien & Lin, 2004). In particular, pickling techniques using salt have the longest history and have been used globally (Li & Hsieh, 2004; Behera et al., 2020). Pickling processes using salt can be categorized into three groups: pretreatment, safe lactic acid (Lac) fermentation, and salt stock preparation. The pretreatment process reduces the water content of the raw materials through the dehydration effect of salt and softens the texture of the raw materials. During the pretreatment process, the juice from the pickled raw materials lowers the concentration of salt in the brine. The diluted brine is thus unable to suppress the growth of undesirable microorganisms for long-term pickling. Therefore, pretreatment is used as an independent short-term process, or as part of a long-term process with an initially high salt content or with the continuous addition of salt (Uthpala et al., 2019). Korean kimchi uses an independent pretreatment process before pickling in spicy brine (Jung et al., 2011). The fermentation process is mainly performed by lactic acid bacteria (LAB) that are relatively resistant to salt. The typical salt content for Lac fermentation is 2–5% to repress the growth of undesired microorganisms (Behera et al., 2020). Lac produced by fermentation decreases the pH of the brine, which reduces the growth of acid-sensitive microorganisms, further enhancing the storability of the product. The salt stock preparation process also uses high salinity brine to increase storability. The salinity of the brine can be up to 20%, where limited halotolerant microbes can survive. Therefore, salt stock preparation is aimed at preservation of vegetables rather than fermentation (Behera et al., 2020). Pickling processes are still very important and of worldwide interest for researchers.

**Table 1:** Brief summary of recent studies on microbiota involved in production of brinefermented vegetables.

Pickle name	Raw material	Pickling solution(s)	Final salinity	Fermentation time	Predominant genus in final microbiota	Reference
Sauerkraut	Cabbage	Brine	2.3%	14d	*Lactobacillus*	Plengvidhya et al. (2007)
	Cabbage	Brine	2.25%	14d	*Leuconostoc*, *Lactobacillus*	Zabat et al. (2018)
Cucumber pickle	Cucumber	Brine	6%	14d	*Lactobacillus*	Pérez-Díaz et al. (2020)
Kimchi	Chinese cabbage	Brine with various spices and fermented seafood	N.E.	29d	*Leuconostoc*, *Lactobacillus*, *Weissella*	Jung et al. (2011)
	Chinese cabbage	Brine with various spices	N.E.	100d	*Lactobacillus*	Jeong et al. (2013)
	Chinese cabbage	Brine with various spices	0 and 5%	50d	*Leuconostoc*	Seo et al. (2018)
Paocai	Cabbage	Brine with various spices	6%	7d	*Lactobacillus*	Xiao et al. (2018)
	Cabbage	Brine	3%	10d or 20d	*Lactobacillus*	Wang et al. (2020)
Suancai	Chinese cabbage	Brine	1%	30d	*Lactobacillus*	Yang et al. (2014)

**Notes.**

N.E. not evaluated d days

The most extensively studied pickles are sauerkraut (Plengvidhya et al., 2007; Zabat et al., 2018), cucumber pickles (Pérez-Díaz et al., 2017; McMurtrie et al., 2019; Pérez-Díaz et al., 2020), Korean kimchi (Jung et al., 2011; Jeong et al., 2013; Hong et al., 2016; Lee et al., 2017; Seo et al., 2018), and Chinese paocai (Xiao et al., 2018; Liu et al., 2019; Wang et al., 2020) and suancai (Yang et al., 2014; Wu et al., 2015) that are produced through fermentation processes. The microbiota of these pickles during the production have been previously studied using culture dependent and independent methods (Table 1). Although the microbiota in each type of pickle are not identical, almost all studies reported that the predominant genus was *Lactobacillus* or *Leuconostoc*, regardless of the pickling time. The studies of Korean kimchi revealed that pickling salinity is a key factor deciding which genus is predominant under the fermentation conditions; the lower the salinity, the higher the relative abundance of *Leuconostoc* (Lee et al., 2017). The changes in concentrations of amino acids and organic acids during pickle fermentation were also studied as they relate to taste and flavor. The main fermentation product is Lac produced by LAB. Heterolactic fermentation LAB produce other various organic acids such as acetic and propionic acid. LAB may also increase amino acid concentrations by excreting peptidase that digests proteins, producing amino acids (Liu et al., 2010). Different amino acids are present in different types of pickles, possibly because of the difference in the microbial community during pickle production (Wu et al., 2015).

Finding the relationship between microbiota and metabolites is a powerful approach to understand the dynamics of metabolites during pickle production and to identify the key bacteria necessary for desirable nutrient and sensory contents. This approach has been successful under Lac fermentation conditions (Jeong et al., 2013), and the dynamics of LAB composition correspond to Lac concentrations during pickle production (Yang et al. , 2016). The relative abundance of *Lactobacillus* is negatively correlated with the concentrations of amino acids such as methionine, tyrosine, lysine and arginine (Liu et al., 2020), and the relative abundance of *Leuconostoc* is positively correlated with the content of acetic acid, butyric acid, cysteine, glycine, isoleucine (Ile) and leucine (Leu) (Xiao et al., 2018). However, the relationship between the microbiota and metabolites in pickles has not been fully evaluated during pretreatment and salt stock preparation. Further studies can illustrate the thorough science of pickle production that has developed since ancient times.

The present study aimed to clarify the changes in microbial composition and concentrations of free amino acids and organic acids by focusing on the processes of independent pretreatment and salt stock preparation that have previously been overlooked. Compared with conditions for Lac fermentation, the pretreatment process uses a similar salt concentration but in the short term. In contrast, salt stock preparation uses a long-term process similar to that for the Lac fermentation process but with a high salt concentration. The results obtained from this study will help to explain the effects of salinity and pickling time on the microbiota as well as the consequent effects of the changed composition of the microbiota on metabolites by comparing the results from the different processing conditions. This will provide valuable knowledge for improving pickle production. Samples were collected from commercial Japanese manufacturers of leafy pickles made from Brassicaceae vegetables. The microbiota were analyzed using 16S rRNA gene sequencing, and 24 free amino acids and organic acids were quantified by liquid chromatography-mass spectrometry (LC-MS). Comparative analysis was used to show that pretreatment of raw materials (short-term pickling at a moderate salinity) results in a microbiota composition more aerobic than that of Lac fermentation. Also, salt stock preparation (long-term pickling at a high salinity) increases the production of branched-chain amino acids (BCAA) by halophilic bacteria, which can be caused by the coenzyme preference of a key enzyme in the BCAA biosynthetic pathway of halophilic bacteria. These results will provide fundamental knowledge on pickling production, which will help to improve the quality of pickles. Also, the results will expand the possibility to produce the pickles with the desired metabolites by changing the pickling conditions to increase the abundance of target bacteria.

## Materials & Methods

### Overview of experimental program

**Figure 1:**
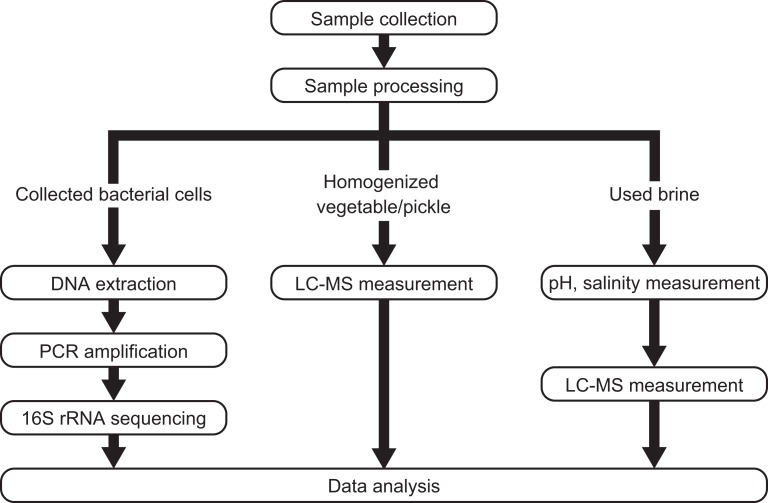
Schematic map of the experimental procedures used in this study. LC-MS, liquid chromatography-mass spectrometry.

A schematic map of the experimental procedures is shown in Fig. 1. Briefly, samples of the raw vegetables, pickles and used brine were collected. Bacterial cells were obtained from all samples by centrifugation then their composition was analyzed. The concentrations of free amino acids and organic acids were determined in the samples of homogenized vegetables and pickles, and the used brine. The changes in the pH and salinity of the process were estimated from the used brine samples. The data were then analyzed statistically.

### Sample collection and processing

Samples of traditional Japanese leafy pickles made from Brassicaceae vegetables were provided by eight commercial manufacturers (Manufacturer A to H). According to the manufacturer’s processes, the initial raw vegetable (Group I), samples from an independent pretreatment process (Group P), or samples from the salt stock preparation process (Group S) were provided. The samples from the pretreatment process and salt stock preparation process included pickled vegetables and used brine (Table 2). The average period for pretreatment was five days, and the average period for the stock preparation process was five months. The pickles were produced between January 2017 and February 2018. The brine samples were used for pH and salinity measurement. Bacteria for DNA extraction were collected from all samples. Raw and pickled vegetables were cut up under aseptic conditions to provide a total surface area of approximately 140 cm^2^, then the vegetables were washed vigorously in 10 ml sterile 0.85% sodium chloride solution for 15 s. The debris was removed from the washing liquid by passing it through a 40 µm filter. The filtered solution was centrifuged at 8,000 g and 4  °C for 10 min, the bacterial cells were collected, and the supernatant was discarded. The cells were collected from the residual brine samples using the same methods of filtering and centrifugation. After cell collection, all samples were stored at −20 °C for DNA extraction and further analysis.

**Table 2:** Samples used in this study.

*Group* / material	Number of samples
*Initial raw vegetable (Group I)*	
Raw vegetable	8
*Pretreatment (Group P)*	
Processed vegetable	4
Used brine	4
**Total**	**8**
*Salt stock preparation (Group S)*	
Processed vegetable	4
Used brine	4
**Total**	**8**

### DNA extraction and 16S rRNA gene sequencing

The collected cells were resuspended in lysozyme (1 g/l, Wako Pure Chemicals, Tokyo, Japan) in tris(hydroxymethyl)aminomethane-ethylenediaminetetraacetic acid (Tris-EDTA) buffer and incubated at 37 °C for 5 min. The DNA was extracted from the processed cells using a Genomic DNA Clean and Concentrator kit (Zymo Research, Irvine, CA, USA). The V3–V4 region of the 16S rRNA gene was PCR-amplified from the extracted DNA using universal primers with Illumina MiSeq (Illumina, San Diego, CA, USA) barcode and adaptor sequences (Mori et al., 2014). The PCR conditions were as follows: one initialization cycle at 94 °C for 30 s, followed by 30 cycles of denaturation at 94 °C for 15 s, annealing at 50  °C for 30 s, and extension at 72 °C for 1 min, then a final extension at 72 °C for 5 min. The size and concentration of the amplified DNA were analyzed using an Agilent DNA High Sensitivity DNA Kit and an Agilent 2100 Bioanalyzer (Agilent Technologies, Santa Clara, CA, USA), and equimolar amounts of DNA from each sample were loaded into the Illumina MiSeq. The raw fastq files for the 24 samples were deposited in the DDBJ (DNA Data Bank of Japan) under the DRA accession number DRA008404 and were associated with the BioProject titled “Microbiome in Japanese Three Major Leafy Pickles” under the BioProject accession number PRJDB8289.

### Measurement of salinity and concentrations of free amino acids and organic acids

The salinity of the used brine was measured with a LAQUAtwin-Salt-22 ion meter (Horiba, Kyoto, Japan) in at least duplicate. The samples for measuring the concentrations of free amino acids and organic acids by LC-MS were prepared as follows: the raw vegetables and pickles were roughly chopped and approximately 1 g aliquots were homogenized in an equivalent amount (w:w) of ultra-pure water using a high-power homogenizer (ASONE, Tokyo, Japan) at 3 000 rpm for 5 min on ice. The homogenate was centrifuged at 20 400 g and 4 °C for 5 min, and the supernatant was retained for analysis. Used brine samples were also centrifuged at 20 400 g and 4  °C for 5 min, and 40 µl supernatant was mixed with 10 µl internal standard solution (methionine sulfone, 5 g/l). Next, 175 µl deproteinization solution (methanol and chloroform, 2.5:1, v:v) was added and mixed vigorously. The mixture was incubated with vigorous shaking at 37  °C for 30 min, and centrifuged at 20 400 g and 4  °C for 5 min. Then, 200 µl supernatant was mixed with 88 µl ultra-pure water and centrifuged at 20 400 g and 4 °C for 5 min. Finally, 200 µl supernatant was mixed with 100 µl ultra-pure water passed through a 0.45 µm filter to remove the insoluble particles. The final aqueous solutions were analyzed using a LCMS-8050 system (Shimadzu, Kyoto, Japan) with a Discovery HS F5-3 column (Sigma-Aldrich, St Louis, MO, USA). The analysis was performed using the Primary Metabolite Ver. 2 method package (Shimadzu). The conditions for liquid chromatography (Optima LC/MS, Fisher Chemical, Waltham, MA, USA) were as follows: mobile phases, solution A, 0.1% formic acid in water, solution B, 0.1% formic acid in acetonitrile; flow rate, 0.25 ml/min with gradient mode; gradient program, 100% solution A from 0 to 2 min, 100%–75% solution A from 2 to 5 min, 75%–65% solution A from 5 to 11 min, 65%–5% solution A from 11 to 15 min and 5% solution from 15 to 20 min; and column oven temperature, 40 °C. The conditions for mass spectrometry were as follows: ionization mode, electrospray ionization; analysis mode, multiple reaction monitoring; nebulizer gas flow rate, 3.0 l/min; drying gas flow rate, 10 l/min; heating gas flow rate, 10 l/min; interface temperature, 300 °C; desolvation line temperature, 250  °C; and heat block temperature, 400 ° C. An external standard was used to calculate the concentration of the compound in mg/l. An internal standard was used to estimate the dilution rate and experimental error. The concentration of each metabolite was transformed into mg/kg based on the sample weight. In the case of homogenized solid samples, the weight was calculated based on an assumed density of 1 g/ml. In the case of liquid samples, the weight was calculated based on the density, which was determined by weighing 1 ml of the sample in at least duplicate. The statistical significance of differences between the mean values of salinity and the concentrations of the free amino acids and organic acids from two groups out of the I, P and S groups was tested repeatedly using the non-parametric Wilcoxon rank sum test (*P* < 0.05). This test enabled the detection of any significant difference in a single variant dataset without assuming that the data was normally distributed. The Bonferroni correction was applied to avoid any false detection of significant differences due to multiple testing.

### Processing 16S rRNA gene sequence data for microbiota analysis

The reads containing N(s) and phiX reads, identified by bowtie2 (version 2.1.0) (Langmead & Salzberg, 2012), were removed. Unpaired reads were deleted using in-house scripts. The processed reads were filtered, denoised, and merged using the dada2 functions (Callahan et al., 2016, p. 2) in the R program (version 1.8). In dada2, the forward and reverse reads were truncated to 270 bp and 220 bp, respectively, and the primer sequences were trimmed. Reads with a labelled Phred score <5 at the first nucleotide or an expected error >2 for a forward read and >5 for a reverse read were removed (Edgar & Flyvbjerg, 2015). After the remaining reads were denoised and merged, an amplicon sequence variant (ASV) table (Callahan, McMurdie & Holmes, 2017) was constructed, and any chimeric reads were removed. The resulting ASV table and reads were imported into QIIME2 (version 2018.11) for further analysis (Bolyen et al., 2018). Default values were used for all optional parameters in the QIIME2 commands. Taxa were assigned to ASVs at the genus level using the Greengenes (version 13.8) database (McDonald et al., 2012), and ASVs for mitochondria and chloroplasts were removed. The reads from genera with average total abundance less than 0.5% were categorized as “Others.” The similarity of the microbiota at the genus level was investigated by principal component analysis (PCA) using R. Beta diversity was analyzed using a built-in function of QIIME 2. Reads from all samples were rarefied to 10 477, which was the smallest read in the dataset, and the distance of the microbiota was calculated using the Bray–Curtis method at the genus level. Any significant differences in microbiota between two groups out of the I, P and S groups were determined using the pairwise permutational multivariate analysis of variance (PERMANOVA) test that enables the detection of significant differences in a multiple variant dataset without assuming a normal distribution. The Benjamini–Hochberg procedure for controlling the False Discovery Rate was applied to avoid any false detection of significant differences due to multiple testing (*q* <0.05).

### Data analysis for estimating metabolic profiles of microbiota

PICRUSt analysis was performed to evaluate the abundance of metabolic pathways in the microbiota and the contribution of each genus to the pathway abundance using stand-alone PICRUSt2 (version 2.1.4 beta) software (Douglas et al., 2019). Default values for optional parameters were used. Based on MetaCyc classification (Caspi et al., 2018), the pathways from glycolysis, tricarboxylic acid (TCA) cycle, fermentation of pyruvate, fermentation to lactate, alanine biosynthesis, Ile biosynthesis, Leu biosynthesis, valine (Val) biosynthesis, and BCAA biosynthesis were selected. Engineered pathways and pathways from specific genera were excluded. Significant differences in the relative abundance of each pathway among sample groups were tested using the Wilcoxon rank sum test (*P* < 0.05) with Bonferroni correction for multiple testing. The total contribution of the genus to the relative abundance of the pathways was calculated, and the genera below the top 10 were categorized as “Others.” Further statistical analysis was performed to detect correlations between the relative abundance of genera and the concentrations of free amino acids and organic acids. Genera with a total average of less than 0.5% were excluded. Significant correlations were determined by the Pearson method using R (*P* < 0.05).

## Results

### Characterization of pretreatment and salt stock preparation samples

The pH and salinity of the used brine samples was measured. The pH of used brine from Group P and S was almost same (the average ± standard deviation; Group P, 7.01 ± 0.47; Group S, 6.45 ± 0.59). The average and standard deviation of the salinity of Group P were 5.3% ± 1.0%, and for Group S they were 17.8% ± 0.2% (Table S1 for raw data). The difference in the salinity between Group P and S was statistically significant. Combined with the information provided by the manufacturers, samples in Group P were salted to relatively low salinity for short term, and samples in Group S were salted to high salinity for long term.

### Difference in microbiota between the process groups

**Figure 2:**
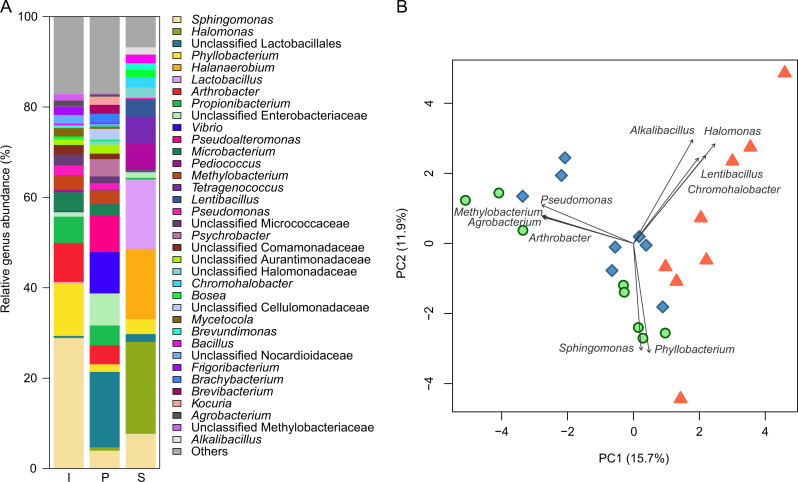
Analysis of microbiota at the genus level during each fermentation process. (A) Composition of microbiota. (B) Scatter plot of principal component analysis. Genera with a total relative abundance of less than 0.5% are categorized as “Others.” I and green circles, initial raw vegetable group (*n* = 8); P and blue diamonds, pretreatment group (*n* = 8); S and orange triangles, salt stock preparation group ( *n* = 8). The 10 most abundant genera were based on the length of the loading vector in the scatter plot.

The analysis of 16S rRNA gene sequences indicated differences in the microbiota during pickle production. In total, 4,635 560 reads were obtained from 24 samples after the mitochondria- and chloroplast-associated reads were removed. The reads represented 4,368 ASVs and 742 genera based on the Greengenes database annotation. The average composition of the genera in each process group is shown in Fig. 2A (Fig. S1 for the composition of genus for each sample). The genus *Sphingomonas* comprised the largest proportion, about 30%, of Group I, followed by *Phyllobacterium* and *Arthorobacter*. These three genera, which are often found in soil and the natural environment, formed 49% of the microbiota of Group I. An unclassified genus of Lactobacillales, an order of LAB, comprised 17% of Group P. In addition, *Pseudoalteromonas* and *Vibrio*, often found in moderately saline environments, formed a relatively high proportion of Group P microbiota. The halophilic genera *Halomonas* and *Halanaerobium* constituted approximately 36% of the bacterial population, and *Lactobacillus* constituted approximately 15% in Group S. There were fewer genera in Group S than in Groups I and P. The scatter plot from PCA (Fig. 2B) showing the pattern of sample groups indicates that the microbiota was changed by the process. A pairwise statistical test using PERMANOVA indicated that the microbiota of all three groups were significantly different from each other (*q*-value: I vs. *P*, 0.003; *I* vs. S, 0.012; *P* vs. S, 0.012).

### Differences in concentrations of free amino acids and organic acids between the groups

**Figure 3:**
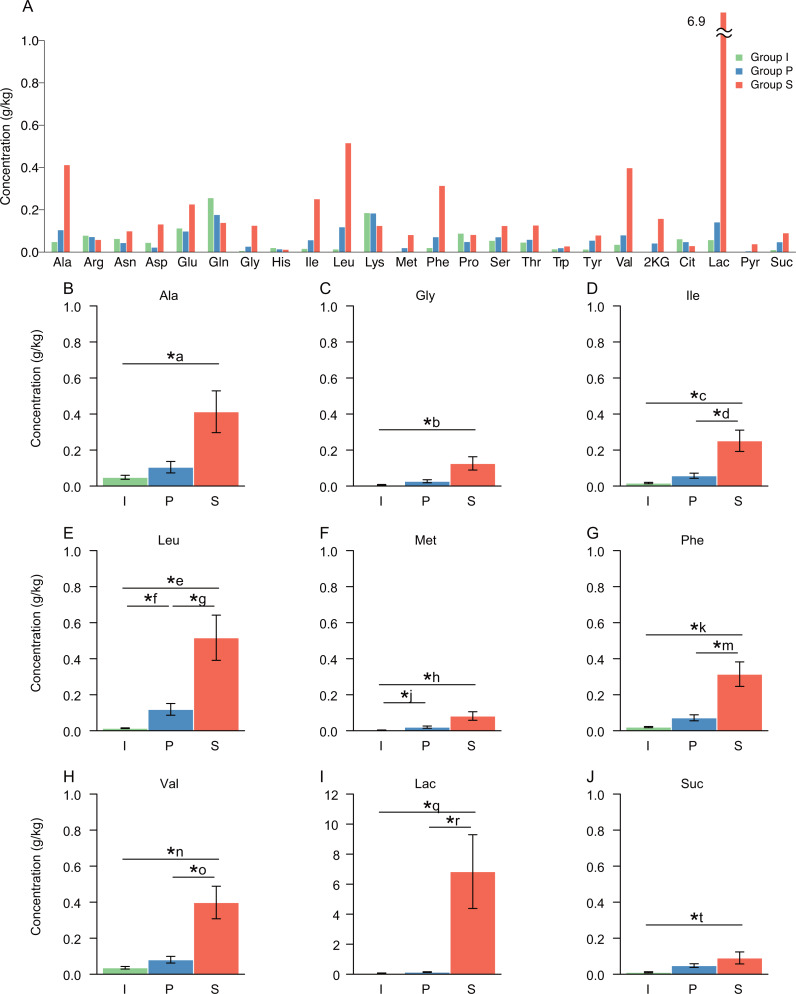
Concentrations of free amino acids and organic acids. (A) All metabolites measured. (B) Metabolites with significant difference in average concentrations among the processes. Error bars indicate the standard error. *, significant difference (*P* < 0.05) with Bonferroni correction for multiple testing. I, initial raw vegetable group (*n* = 8); P, pretreatment group (*n* = 8); S, salt stock preparation group (*n* = 8). Ala, alanine; Arg, arginine; Asn, asparagine; Asp, aspartic acid; Glu, glutamic acid; Gln, glutamine; Gly, glycine; His, histidine; Ile, isoleucine; Leu, leucine; Lys, lysine; Met, methionine; Phe, phenylalanine; Pro, proline; Ser, serine; Thr, threonine; Trp, tryptophan; Tyr, tyrosine; Val, valine; 2KG, 2-ketoglutaric acid; Cit, citric acid; Lac, lactic acid; Pyr, pyruvic acid; Suc, succinic acid. *P*-values for metabolites with significant difference were as followed: *a, 0.001; *b, 0.001; *c, 0.0002; *d, 0.002; *e, 0.0002; *f, 0.010; *g, 0.007; *h, 0.010; *j, 0.010; *k, 0.0002; *m, 0.001; *n, 0.0002; *o, 0.001; *q, 0.0002; *r, 0.0002; *t, 0.005.

A significantly different microbiota should affect the amount of amino acids and organic acids in the samples. The LC-MS analysis quantified 24 metabolites of free amino acids and organic acids (Fig. 3A, Table S2 for raw data). The concentrations of several metabolites were higher in Group S than in the other groups. Salt stock preparation process was not recognized as a fermentation process, but a considerable amount of Lac was produced: on average, 118 times higher in Group S than in Group I and 48 times higher in Group S than in Group P. Group S showed significantly higher concentrations of Ile (*P*-value: *I* vs. *S*, 0.0002; *P* vs. *S*, 0.002), Leu (*P*-value: *I* vs. *S*, 0.0002; *P* vs. *S*, 0.0007), phenylalanine (*P*-value: *I* vs. *S*, 0.0002; *P* vs. *S*, 0.001), Val (*P*-value: *I* vs. *S*, 0.0002; *P* vs. *S*, 0.001) and Lac (*P*-value: *I* vs. *S*, 0.0002; *P* vs. *S*, 0.0002) than the other two groups (Fig. 2B and Table S2). In contrast, Group P showed significantly higher concentrations of only two metabolites, Leu (*P*-value: I vs. P, 0.01) and methionine (*P*-value: I vs. P, 0.01) than Group I (Fig. 3B and Table S2), which indicates that the concentrations of the measured metabolites in Group P were generally equivalent to the concentrations in Group I.

### The effect of the microbiota in Group S on concentrations of free amino acids and organic acids

**Figure 4:**
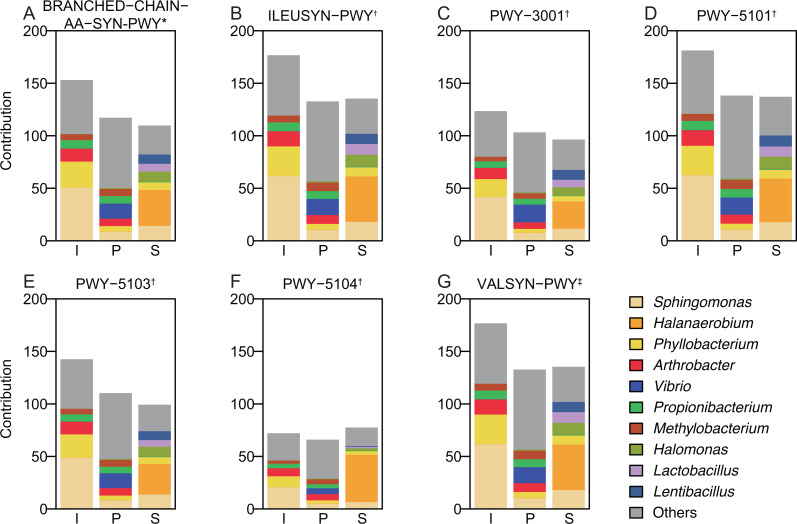
Contribution of each genus to the abundance of pathways related to branched-chain amino acid biosynthesis (A-G). Those genera not representing the 10 most abundant were categorized together as “Others.” I, initial raw vegetable group (*n* = 8); P, pretreatment group (*n* = 8); S, salt stock preparation group (*n* = 8). *, a pathway of branched-chain amino acid biosynthesis; † , pathways of isoleucine biosynthesis; ‡ , a pathway of valine biosynthesis. Contributions to pathway abundances were calculated by multiplying the relative abundance by the copy number of the gene(s).

A further analysis was used to identify the relationship between microbiota and metabolites. Among the metabolites that had significantly different concentrations when comparing Group S and the other two groups, Ile, Leu, Val, and Lac are derivatives of pyruvate based on the generic metabolic pathway (Table S3). Therefore, pyruvate metabolism is expected to be a key in explaining the changes in metabolite concentrations caused by the microbiota. PICRUSt analysis revealed the abundance of pathways related to pyruvate metabolism such as glycolysis, the TCA cycle, and pyruvate fermentation, as well as biosynthetic pathways of Lac, alanine and BCAA (Ile, Leu, and Val; Table 3; Fig. S2 with pathway description), with 25 pathways detected overall. The statistical analysis showed that the abundance of the standard TCA cycle (TCA) was significantly lower in Group S than in Group I (*P*-value: *I* vs. *S*, 0.007). Also, the abundances of all the Lac biosynthetic pathways were higher in Group S than in Group I (Table 3). These results indicate that the microbiota in Group S comprised anaerobic microbes. In contrast, the abundances of pathways for fermentation to lactate in Groups I and P were relatively similar, which reflects the lower concentration of Lac in those groups. The abundance of glycolysis pathways was similar in all groups, possibly because glycolysis is a fundamental pathway for all bacteria. Unexpectedly, the abundances of amino acid biosynthesis pathways in Group S were not significantly different from those in the other groups, which was not consistent with the results from LC-MS analysis, indicating that the changes in metabolite concentrations were caused by factors other than pathway abundance. PICRUSt2 contribution analysis was used to identify the genera causing qualitative differences in pathway abundance. Group-specific genera made the highest contribution to the amino acid biosynthetic pathways evaluated (Fig. 4): *Sphingomonas* or *Arthrobacter* in Group I, *Vibrio* in Group P, and *Halanaerobium* in Group S. *Halanaerobium* may play an important role in the production of Ile, Leu, and Val under high salinity conditions. A correlation analysis confirmed the relationship between the genera and metabolite production. The relative abundances of two genera, *Halanaerobium* and *Chromohalobacter*, were significantly correlated with the concentrations of Ile, Leu, and Val (Table 4), with higher correlation coefficients between *Halanaerobium* and metabolite concentrations (*r* = 0.71–0.84) highlighting the possible relationship.

**Table 3:** The average pathway abundance analysed by PICRUSt.

Category^*^	Pathway ID^*^	Average relative abundance (%)		
		I	P	S
Glycolysis	ANAGLYCOLYSIS-PWY	0.62	0.69	0.77
	GLYCOLYSIS	0.47	0.64	0.70
	PWY-5484	0.47	0.64	0.68
TCA cycle	P105-PWY	0.67^a^	0.57^b^	0.40
	PWY-5913	0.50	0.46	0.37
	PWY-6969	0.69^a^	0.58^b^	0.41
	PWY-7254	0.36	0.38	0.22
	TCA	0.74^a^	0.62^b^	0.42^b^
Fermentation of pyruvate	CENTFERM-PWY	0.02	0.02	0.04
	FERMENTATION-PWY	0.20^a^	0.46^b^	0.30
	P108-PWY	0.55^a^	0.30^b^	0.32
	P161-PWY	0.08^a^	0.24	0.56^b^
	PWY-6588	0.04	0.05	0.11
Fermentation to Lactate	P122-PWY	0.12	0.15	0.31
	P124-PWY	0.21	0.19	0.42
	P461-PWY	0.06^a^	0.13^b^	0.26^b^
	PWY-5100	0.18	0.21	0.64
Ala biosynthesis	PWY0-1061	0.31	0.52	0.43
BCAA biosynthesis	BRANCHED-CHAIN-AA-SYN-PWY	0.80^a^	0.64^b^	0.71
Ile biosynthesis	ILEUSYN-PWY	0.91^a^	0.72^b^	0.88
	PWY-3001	0.67^a^	0.57^b^	0.64
	PWY-5101	0.95^a^	0.75^b^	0.88
	PWY-5103	0.75^a^	0.61^b^	0.64
	PWY-5104	0.40	0.43	0.49
Val biosynthesis	VALSYN-PWY	0.91^a^	0.72^b^	0.88

**Notes.**

* Category and Pathway ID were from the MetaCyc database (https:/metacyc.org/).

I initial raw vegetable group P pretreatment group S salt stock preparation group Ala alanine BCAA branched-chain amino acid Ile isoleucine Val valine

Different superscript letters in a row indicate a significant difference between the mean values (*P* < 0.05).

**Table 4:** Significant correlations (*P* < 0.05) between the relative abundance of the genus and the concentrations of isoleucine, leucine or valine.

Genus name	Amino acid	Correlation coefficient	*P*-value
*Halanaerobium*	Isoleucine	0.84	0.0001
	Leucine	0.71	0.002
	Valine	0.84	0.00004
*Chromohalobacter*	Isoleucine	0.57	0.022
	Leucine	0.52	0.038
	Valine	0.52	0.038

## Discussion

This study has focused on two processes for producing pickles, pretreatment and salt stock preparation, with the intent of clarifying the effects of the pickling conditions on microbiota and metabolites. The average salinity of the pretreatment group (Group P) was 6%, which is similar to the conditions in previously reported fermentation process (Table 1). Microbiota can be dominated by LAB within three days under moderate salinity conditions (Pérez-Díaz et al., 2020). However, LAB were relatively abundant but not dominant in Group P. The predominance of aerobic genera in Group P suggested that the pickling environment during the pretreatment process was not as anaerobic as the environment during the fermentation process. This may be because strictly anaerobic conditions are not required for pretreated vegetables which will be re-pickled in the final fermentation environment. The results from the pretreatment process illustrate the effect of inadequately anaerobic conditions on the dominance of LAB in pickle microbiota. In addition, *Vibrio*, a potentially unfavorable genus for food production, was relatively predominant. This result corresponds to a previous report indicating that salt-cured food can be contaminated with undesired halotolerant microbes (Kim, Cho & Rhee, 2017). Because *Vibrio* is a halotolerant and facultative anaerobic genus, it is difficult to eliminate this genus by ambient salinity and anaerobic conditions. Based on the results that the concentrations of free amino acids and organic acids remained the same as in the raw materials during the pretreatment process and pH was close to neutral, both anaerobic and low pH environmental conditions, resulting from the fermentation process, should be necessary for safe pickle production under ambient salinity.

The salt stock preparation process did not cause the microbiota to be dominated by LAB but by a mixture of *Lactobacillus* and the halophilic bacteria *Halomonas* and *Halanaerobium*, which was different from the results of previous studies under Lac fermentation conditions (Table 1). Only a limited number of LAB genera can grow under high salinity conditions, which would suppress the dominance of LAB in the salt stock preparation process. The results of the quantification and statistical tests of the metabolites showed that the concentrations of five of the 24 metabolites differed significantly between Group S and the other groups. Of these, four metabolites (Ile, Val, Leu (BCAA), and Lac) are derived from pyruvate based on the generic metabolic pathway (Fig. S2). Generally, under conditions where aerobic respiration is not available, the TCA cycle is suppressed and pyruvate is used to re-oxidize excess NADH with lactate dehydrogenase to produce Lac. The PICRUSt analysis showed that the TCA cycle was reduced and that Lac biosynthesis was enhanced in Group S, indicating that pyruvate is used to produce Lac under the anaerobic conditions in the salt stock preparation process. It has to be noted that there was a discrepancy between Lac concentration and pH in salt stock preparation samples. Although Lac concentration in Group S was significantly higher than that in Group P, pH values of Group P and S were almost same. Further analysis is needed to clarify the reason for the results but ammonium produced by amino acid catabolism might be the factor to neutralize Lac in salt stock preparation process.

The abundance of the pathway for BCAA biosynthesis, which also originates from pyruvate, remained the same even though BCAA concentrations were significantly increased. The contribution analysis of pathways using PICRUSt showed that the genus with highest contribution was different in each group, which suggests that the pathways had qualitative differences in enzyme activity or substrate affinity and/or selectivity. In Group S, *Halanaerobium* had the highest contribution to all pathways for BCAA biosynthesis. Correlation analysis also indicated a significant correlation between the relative abundance of *Halanaerobium* and the production of BCAA, suggesting that the specific feature of *Halanaerobium* metabolism resulted in an increase in the pyruvate-family amino acids during the salt stock preparation process. This proposed mechanism is different from that in the fermentation process where LAB produce extracellular peptidase to degrade soluble proteins and increase free amino acids (Liu et al., 2010). The mechanism of how BCAA concentration is increased by *Halanaerobium* can be explained by the enzymatic character of ketol-acid reductoisomerase (KARI). KARI is a key enzyme in the Ile and Leu/Val biosynthetic pathways (Chen et al., 2018) and is known to use NADPH as a coenzyme (Fig. S2). Recently, KARI has been reported to enhance the productivity of BCAA by changing the coenzyme selectivity from NADPH to NADH (Brinkmann-Chen, Cahn & Arnold, 2014). Several species of thermophilic, acidophilic and halophilic bacteria have been found to have KARI which prefers NADH to NADPH. The selectivity of NADH can be caused by four acidic amino acid residues on KARI. The amino acid sequence of KARI in *Halanaerobium congolens* includes aspartic acid as the second key residue, and the same residue in *Desulfococcus oleovorans* has been shown to prefer NADH as a coenzyme (Fig. S3) (Brinkmann-Chen, Cahn & Arnold, 2014). Some species of *Halanaerobium* including *H. congolens* may have KARI that uses NADH, which indicates that the BCAA biosynthetic pathway as well as lactate dehydrogenase can re-oxidize NADH. This feature may provide an advantage for maintaining the metabolic redox balance under anaerobic conditions. However, this proposed mechanism is still hypothetical. Isolating the *Halanaerobium* strain from the samples would allow the investigation of enzymatic profiles and physiological features to clarify the mechanisms between *Halanaerobium* and BCAA production. The results from such a future study might enable the BCAA content of pickles to be improved by increasing the abundance of specific species of *Halanaerobium* or bacteria which have the same metabolic features as *Halanaerobium*.

It should be noted that the present study is not completely comprehensive because the interactions of other bacteria present in the microbiota during pickle production process have not yet been evaluated. This research topic needs further investigation in future studies to clarify the relationship between the various combinations of bacteria and metabolites to provide a better understanding of the science underlying vegetable fermentation.

## Conclusions

This study has revealed the effects of processing conditions on the microbiota and concentrations of free amino acids and organic acids in pickled vegetables. Pretreatment using a moderate salinity with short-term pickling led to conditions which were not completely anaerobic thus allowing an undesired genus, *Vibrio*, to become relatively abundant. The experimental results also emphasized the importance of the combination of anaerobic and acidic conditions for assuring safer pickle production. The salt stock preparation conditions using high salinity with long-term pickling caused halophilic bacteria to become predominant. Further analysis has indicated possible relationships between *Halanaerobium* and BCAA production through a hypothetical metabolic mechanism derived from the preference of coenzyme as a key enzyme in the BCAA biosynthetic pathway. Future studies on identifying bacteria which can produce BCAA under salt stock preparation conditions will provide more precise knowledge on the relationships between microbiota and metabolites during pickle production. The present study has provided an insight into how controlling pickling conditions can change the abundance of bacteria and thus improve the metabolite composition of pickled vegetables.

##  Supplemental Information

10.7717/peerj.11123/supp-1 Supplemental Information 1 Salinity and pH of brine sample Sample names are defined as Manufacture ID (A-H) - process ID (P, S) [ sample type (b)]. Process ID: P, pretreatment; S, salt stock preparation. Sample type: b, brine.

10.7717/peerj.11123/supp-2 Supplemental Information 2 Amino acids and organic acids concentrations determined by LC-MS Sample names are defined as Manufacture ID (A-H) - process ID (I, P, S) [ sample type (p, b)]. Process ID: I, initial material; P, pretreatment; S, salt stock preparation. Sample type: p, pickle; b, brine. Sample types are applied to samples from pretreatment and salt stock preparation process.

10.7717/peerj.11123/supp-3 Supplemental Information 3 Full description of the pathways analyzed by PICRUSt *, Category and Pathway ID and description were derived from the MetaCyc database (https://metacyc.org/). I, initial raw vegetable group; P, pretreatment group; S, salt stock preparation group; BCAA, branched-chain amino acid. Different superscript letters in a row indicate a significant difference between the mean values (*P* < 0.05).

10.7717/peerj.11123/supp-4 Supplemental Information 4 Bacterial composition at the genus level in each sample Genera with a total relative abundance of less than 0.5% are categorized as “Others.”

10.7717/peerj.11123/supp-5 Supplemental Information 5 Generic schematic pathway for pyruvate metabolism Arrows indicate the flow of a metabolite. Rounded squares represent the group of pathways indicated. Squares indicate the enzyme related to NAD(P)H metabolism. KARI, ketol-acid reductoisomerase; IPMDH, 3-isopropylmalate dehydrogenase; LDH, lactate dehydrogenase.

10.7717/peerj.11123/supp-6 Supplemental Information 6 Amino acid alignment of ketol-acid reductoisomerase First row, *Desulfococcus oleovorans*; second row, *Halanaerobium congolens*. The alignments were obtained from Protein BLAST (NCBI, https://blast.ncbi.nlm.nih.gov/Blast.cgi. The amino acids shown in red are the critical residues for NADPH/NADH selectivity (Brinkmann-Chen, Cahn & Arnold, 2014).
**Reference** Brinkmann-Chen S, Cahn JKB, Arnold FH. 2014. Uncovering rare NADH-preferring ketol-acid reductoisomerases. *Metab Eng* 26 :17–22.

## Additional Information and Declarations

### Data Availability

The following information was supplied regarding data availability: 16S rRNA sequencing data are available in the DDBJ/EMBL/GenBank databases: DRA008404. Raw data from pH, salt content and LC-MS measurements are available in the Supplementary Files.
