# Supplementary material for: The effects of vegetable pickling conditions on the dynamics of microbiota and metabolites
Source: PeerJ. 2021 Apr 6;9:e11123. doi: 10.7717/peerj.11123 (PMC8034358; doi:10.7717/peerj.11123)
Supplement: Supplemental Information 1 — Sample names are defined as Manufacture ID (A-H) - process ID (P, S) [ sample type (b)]. Process ID: P, pretreatment; S, salt stock preparation. Sample type: b, brine. [file peerj-09-11123-s001.docx]

**Table S1:**

**Salinity and pH of brine sample.**

|  | A-Pb | B-Pb | C-Pb | E-Pb | D-Sb | F-Sb | G-Sb | H-Sb |
| --- | --- | --- | --- | --- | --- | --- | --- | --- |
| Salinity (%) | 4.0 | 7.8 | 5.8 | 3.4 | 18 | 18 | 18 | 17 |
| pH | 5.90 | 7.80 | 6.55 | 7.80 | 7.51 | 5.95 | 4.80 | 7.52 |

Sample names are defined as Manufacture ID (A-H) - process ID (P, S) [ sample type (b)]. Process ID: P, pretreatment; S, salt stock preparation. Sample type: b, brine.
