# Supplementary material for: The effects of vegetable pickling conditions on the dynamics of microbiota and metabolites
Source: PeerJ. 2021 Apr 6;9:e11123. doi: 10.7717/peerj.11123 (PMC8034358; doi:10.7717/peerj.11123)
Supplement: Supplemental Information 2 — Sample names are defined as Manufacture ID (A-H) - process ID (I, P, S) [ sample type (p, b)]. Process ID: I, initial material; P, pretreatment; S, salt stock preparation. Sample type: p, pickle; b, brine. Sample types are applied to samples from pretreatment and salt stock preparation process. [file peerj-09-11123-s002.docx]

**Table S2:**

**Amino acids and organic acids concentrations determined by LC-MS.**

| Metabolites | concentrations (mg/kg) | | | | | | | | | | | | | | | |
| --- | --- | --- | --- | --- | --- | --- | --- | --- | --- | --- | --- | --- | --- | --- | --- | --- |
|  | Group I | | | | | | | | Group P | | | | | | | |
|  | A-I | B-I | C-I | D-I | E-I | F-I | G-I | H-I | A-Pp | A-Pb | B-Pp | B-Pb | C-Pp | C-Pb | E-Pp | E-Pb |
| Alanine | 119.0 | 51.8 | 50.0 | 28.6 | 17.1 | 30.0 | 37.2 | 56.2 | 144.9 | 120.2 | 199.2 | 42.9 | 255.6 | 39.5 | 34.4 | 3.2 |
| Arginine | 25.2 | 88.2 | 8.9 | 23.9 | 213.1 | 152.9 | 16.0 | 101.9 | 75.6 | 87.8 | 133.8 | 27.3 | 120.2 | 22.7 | 111.3 | 0.7 |
| Asparagine | 53.6 | 95.7 | 10.1 | 30.0 | 48.8 | 135.5 | 32.6 | 101.8 | 50.5 | 64.8 | 104.2 | 22.9 | 50.8 | 7.4 | 52.0 | 0.0 |
| Aspartic acid | 45.7 | 65.5 | 7.9 | 34.3 | 27.8 | 59.2 | 56.7 | 64.0 | 27.3 | 31.6 | 46.9 | 13.8 | 32.4 | 10.0 | 18.3 | 0.0 |
| Cystine | 5.7 | 12.4 | 7.2 | 11.9 | 0.2 | 1.8 | 6.2 | 5.0 | 13.7 | 8.2 | 15.6 | 1.9 | 8.2 | 1.7 | 0.8 | 0.0 |
| Glutamic acid | 122.1 | 183.8 | 51.9 | 12.9 | 35.9 | 105.3 | 160.2 | 234.5 | 106.7 | 125.3 | 184.4 | 34.0 | 247.7 | 29.0 | 61.3 | 0.8 |
| Glutamine | 462.7 | 539.4 | 14.8 | 173.9 | 76.4 | 51.4 | 111.4 | 621.1 | 232.3 | 463.5 | 388.4 | 60.2 | 176.0 | 32.6 | 62.1 | 0.7 |
| Glycine | 22.2 | 5.1 | 3.8 | 4.7 | 3.9 | 5.3 | 0.8 | 5.8 | 38.2 | 36.3 | 68.6 | 9.9 | 45.2 | 8.8 | 7.2 | 1.3 |
| Histidine | 17.2 | 33.9 | 3.8 | 5.6 | 23.6 | 25.9 | 19.3 | 34.5 | 13.9 | 13.9 | 27.4 | 4.8 | 22.1 | 2.5 | 33.0 | 0.0 |
| Isoleucine | 11.6 | 16.1 | 9.7 | 14.9 | 10.1 | 13.8 | 14.7 | 42.7 | 67.6 | 96.2 | 122.0 | 33.1 | 75.2 | 16.8 | 48.1 | 0.4 |
| Leucine | 5.2 | 9.5 | 18.9 | 13.0 | 21.2 | 12.2 | 10.6 | 21.0 | 152.9 | 190.3 | 254.6 | 52.8 | 200.6 | 46.4 | 51.5 | 0.5 |
| Lysine | 479.5 | 553.1 | 15.3 | 172.0 | 78.7 | 47.3 | 35.1 | 106.0 | 239.3 | 477.4 | 407.6 | 62.5 | 181.1 | 33.9 | 69.7 | 1.0 |
| Methionine | 2.5 | 2.3 | 3.9 | 6.1 | 5.0 | 4.3 | 2.2 | 3.6 | 19.2 | 14.6 | 44.4 | 11.2 | 47.7 | 13.2 | 10.0 | 0.8 |
| Phenylalanine | 7.5 | 21.5 | 10.6 | 15.3 | 33.0 | 26.6 | 16.3 | 32.9 | 82.2 | 104.3 | 141.2 | 43.4 | 109.1 | 25.5 | 68.3 | 0.9 |
| Proline | 8.1 | 20.3 | 25.7 | 13.2 | 10.6 | 10.1 | 141.0 | 480.1 | 33.9 | 43.1 | 169.3 | 13.7 | 46.2 | 6.8 | 79.9 | 0.0 |
| Serine | 80.5 | 44.4 | 17.2 | 26.3 | 38.4 | 58.2 | 69.4 | 103.7 | 94.6 | 101.0 | 168.3 | 28.8 | 75.1 | 21.6 | 83.3 | 0.0 |
| Threonine | 32.4 | 36.8 | 37.2 | 26.6 | 48.8 | 62.6 | 58.3 | 69.3 | 70.2 | 69.1 | 130.6 | 20.9 | 82.6 | 17.2 | 84.9 | 0.7 |
| Tryptophan | 3.6 | 9.6 | 2.6 | 5.6 | 18.9 | 21.5 | 23.3 | 31.0 | 19.2 | 21.5 | 43.3 | 9.0 | 28.7 | 4.4 | 32.2 | 0.2 |
| Tyrosine | 7.3 | 12.3 | 9.3 | 10.8 | 14.4 | 18.0 | 8.9 | 26.2 | 67.1 | 88.9 | 116.4 | 26.3 | 86.5 | 17.9 | 38.3 | 0.5 |
| Valine | 21.4 | 31.2 | 17.4 | 15.5 | 37.0 | 44.7 | 42.9 | 77.0 | 93.6 | 120.7 | 163.8 | 50.6 | 101.7 | 25.9 | 86.4 | 1.6 |
| 2-Ketoglutaric acid | 0.0 | 0.0 | 0.0 | 0.0 | 0.0 | 0.0 | 0.0 | 0.0 | 0.0 | 0.0 | 335.6 | 0.0 | 0.0 | 0.0 | 0.0 | 0.0 |
| Citric acid | 110.7 | 42.9 | 159.9 | 91.1 | 3.4 | 36.3 | 37.4 | 15.6 | 17.7 | 70.5 | 67.9 | 20.9 | 141.8 | 40.0 | 28.7 | 0.0 |
| Lactic acid | 49.4 | 80.2 | 0.0 | 0.0 | 185.3 | 149.6 | 0.0 | 0.0 | 98.9 | 102.8 | 84.6 | 92.2 | 134.5 | 264.3 | 216.0 | 140.5 |
| Pyruvic acid | 1.2 | 0.0 | 0.0 | 0.0 | 0.0 | 0.0 | 0.0 | 0.0 | 0.0 | 0.0 | 0.0 | 10.8 | 0.0 | 27.8 | 0.0 | 0.0 |
| Succinic acid | 31.3 | 2.0 | 11.6 | 6.3 | 4.5 | 13.1 | 8.5 | 7.5 | 66.4 | 83.9 | 45.7 | 29.1 | 67.9 | 20.5 | 69.0 | 1.1 |

(Cont.)

| Metabolites | concentrations (mg/kg) | | | | | | | | *P*-value | | |
| --- | --- | --- | --- | --- | --- | --- | --- | --- | --- | --- | --- |
|  | Group S | | | | | | | | I v.s. P | I v.s. S | P v.s. S |
|  | D-Sp | D-Sb | F-Sp | F-Sb | G-Sp | G-Sb | H-Sp | H-Sb |  |  |  |
| Alanine | 115.5 | 146.5 | 246.5 | 71.0 | 687.5 | 446.9 | 591.2 | 995.5 | 0.279 | 0.001 | 0.028 |
| Arginine | 33.6 | 54.8 | 10.0 | 159.7 | 37.0 | 40.4 | 118.1 | 16.9 | 1.000 | 1.000 | 0.721 |
| Asparagine | 0.0 | 0.0 | 67.1 | 120.4 | 349.6 | 25.9 | 228.1 | 4.8 | 0.505 | 0.773 | 0.720 |
| Aspartic acid | 105.8 | 155.5 | 18.0 | 69.5 | 134.9 | 324.1 | 243.5 | 4.9 | 0.038 | 0.083 | 0.038 |
| Cystine | 1.2 | 1.9 | 6.9 | 2.2 | 9.0 | 1.5 | 5.9 | 0.1 | 1.000 | 0.279 | 0.591 |
| Glutamic acid | 98.9 | 125.7 | 114.8 | 59.6 | 547.0 | 677.5 | 187.8 | 0.9 | 0.798 | 0.574 | 0.328 |
| Glutamine | 112.3 | 154.1 | 161.4 | 166.8 | 213.6 | 106.3 | 201.6 | 0.4 | 0.574 | 0.798 | 0.878 |
| Glycine | 69.8 | 97.1 | 101.5 | 16.9 | 309.3 | 199.8 | 205.7 | 6.6 | 0.021 | 0.001 | 0.028 |
| Histidine | 0.0 | 37.1 | 0.9 | 28.3 | 4.3 | 12.9 | 16.8 | 0.0 | 0.266 | 0.187 | 0.626 |
| Isoleucine | 100.4 | 195.8 | 96.8 | 76.6 | 298.2 | 489.0 | 264.2 | 490.2 | 0.021 | 0.0002 | 0.002 |
| Leucine | 190.5 | 346.1 | 215.2 | 74.0 | 850.2 | 999.9 | 611.8 | 841.8 | 0.010 | 0.0002 | 0.007 |
| Lysine | 120.7 | 154.2 | 168.7 | 54.7 | 195.1 | 108.7 | 196.9 | 0.4 | 0.959 | 0.798 | 0.721 |
| Methionine | 39.6 | 77.7 | 48.8 | 18.4 | 180.0 | 164.0 | 124.3 | 1.6 | 0.010 | 0.010 | 0.038 |
| Phenylalanine | 127.3 | 219.2 | 123.2 | 117.1 | 435.8 | 586.2 | 376.5 | 529.3 | 0.028 | 0.0002 | 0.001 |
| Proline | 7.4 | 8.5 | 115.6 | 32.8 | 184.0 | 87.8 | 223.0 | 1.9 | 0.798 | 0.959 | 0.574 |
| Serine | 83.0 | 117.9 | 51.9 | 105.7 | 204.7 | 154.6 | 279.2 | 0.8 | 0.574 | 0.050 | 0.195 |
| Threonine | 90.0 | 131.4 | 39.2 | 87.8 | 218.3 | 186.1 | 252.2 | 11.3 | 0.505 | 0.038 | 0.065 |
| Tryptophan | 37.3 | 54.8 | 7.6 | 45.3 | 13.4 | 34.4 | 24.7 | 7.5 | 0.523 | 0.083 | 0.328 |
| Tyrosine | 97.1 | 155.2 | 16.5 | 51.9 | 30.4 | 195.1 | 87.3 | 5.2 | 0.021 | 0.021 | 0.574 |
| Valine | 174.3 | 310.0 | 147.5 | 129.8 | 476.8 | 709.8 | 439.7 | 797.7 | 0.065 | 0.0002 | 0.001 |
| 2-Ketoglutaric acid | 0.0 | 0.0 | 0.0 | 0.0 | 0.0 | 686.8 | 580.1 | 0.0 | 1.000 | 0.467 | 0.467 |
| Citric acid | 0.0 | 173.6 | 2.2 | 55.7 | 5.1 | 1.7 | 0.0 | 0.0 | 0.645 | 0.064 | 0.137 |
| Lactic acid | 1765.1 | 2647.9 | 3946.2 | 320.8 | 14463.0 | 17752.7 | 12670.7 | 1121.9 | 0.034 | 0.0002 | 0.000 |
| Pyruvic acid | 0.0 | 127.2 | 32.6 | 0.0 | 45.8 | 47.9 | 53.9 | 0.0 | 0.467 | 0.026 | 0.054 |
| Succinic acid | 23.8 | 24.9 | 32.3 | 28.6 | 174.5 | 236.7 | 193.7 | 8.8 | 0.021 | 0.005 | 0.878 |

Sample names are defined as Manufacture ID (A-H) - process ID (I, P, S) [ sample type (p, b)]. Process ID: I, initial material; P, pretreatment; S, salt stock preparation. Sample type: p, pickle; b, brine. Sample types are applied to samples from pretreatment and salt stock preparation process.
