# Supplementary material for: The effects of vegetable pickling conditions on the dynamics of microbiota and metabolites
Source: PeerJ. 2021 Apr 6;9:e11123. doi: 10.7717/peerj.11123 (PMC8034358; doi:10.7717/peerj.11123)
Supplement: Supplemental Information 3 — *, Category and Pathway ID and description were derived from the MetaCyc database (https://metacyc.org/). I, initial raw vegetable group; P, pretreatment group; S, salt stock preparation group; BCAA, branched-chain amino acid. Different superscript letters in a row indicate a significant difference between the mean values (P < 0.05). [file peerj-09-11123-s003.docx]

**Table S3:**

**Full description of the pathways analyzed by PICRUSt.**

| Category* | Pathway* | | Average abundance (%) | | | *P*-value | | |
| --- | --- | --- | --- | --- | --- | --- | --- | --- |
|  | ID | Description | I | P | S | I vs. P | I vs. S | P vs. S |
| Glycolysis | ANAGLYCOLYSIS-PWY | Glycolysis III (from glucose) | 0.62 | 0.69 | 0.77 | 0.442 | 0.065 | 0.279 |
|  | GLYCOLYSIS | Glycolysis I (from glucose 6-phosphate) | 0.47 | 0.64 | 0.70 | 0.105 | 0.083 | 0.382 |
|  | PWY-5484 | Glycolysis II (from fructose 6-phosphate) | 0.47 | 0.64 | 0.68 | 0.234 | 0.161 | 0.878 |
| TCA cycle | P105-PWY | TCA cycle IV (2-oxoglutarate decarboxylase) | 0.67^a^ | 0.57^b^ | 0.40 | 0.003 | 0.065 | 0.328 |
|  | PWY-5913 | Partial TCA cycle (obligate autotrophs) | 0.50 | 0.46 | 0.37 | 0.328 | 0.083 | 0.442 |
|  | PWY-6969 | TCA cycle V (2-oxoglutarate:ferredoxin oxidoreductase) | 0.69^a^ | 0.58^b^ | 0.41 | 0.003 | 0.021 | 0.328 |
|  | PWY-7254 | TCA cycle VII (acetate-producers) | 0.36 | 0.38 | 0.22 | 0.798 | 0.130 | 0.083 |
|  | TCA | TCA cycle I (prokaryotic) | 0.74^a^ | 0.62^b^ | 0.42^b^ | 0.003 | 0.007 | 0.195 |
| Fermentation of pyruvate | CENTFERM-PWY | Pyruvate fermentation to butanoate | 0.02 | 0.02 | 0.04 | 0.645 | 0.574 | 0.721 |
|  | FERMENTATION-PWY | Mixed acid fermentation | 0.20^a^ | 0.46^b^ | 0.30 | 0.001 | 0.065 | 0.038 |
|  | P108-PWY | Pyruvate fermentation to propanoate I | 0.55^a^ | 0.30^b^ | 0.32 | 0.010 | 0.105 | 0.721 |
|  | P161-PWY | Acetylene degradation (anaerobic) | 0.08^a^ | 0.24 | 0.56^b^ | 0.028 | 0.005 | 0.130 |
|  | PWY-6588 | Pyruvate fermentation to acetone | 0.04 | 0.05 | 0.11 | 0.382 | 0.645 | 0.645 |
| Fermentation to Lactate | P122-PWY | Heterolactic fermentation | 0.12 | 0.15 | 0.31 | 0.721 | 0.959 | 0.959 |
|  | P124-PWY | Bifidobacterium shunt | 0.21 | 0.19 | 0.42 | 0.959 | 0.721 | 0.721 |
|  | P461-PWY | Hexitol fermentation to lactate, formate, ethanol and acetate | 0.06^a^ | 0.13^b^ | 0.26^b^ | 0.010 | 0.005 | 0.195 |
|  | PWY-5100 | Pyruvate fermentation to acetate and lactate II | 0.18 | 0.21 | 0.64 | 0.574 | 0.028 | 0.065 |
| Ala biosynthesis | PWY0-1061 | Superpathway of L-alanine biosynthesis | 0.31 | 0.52 | 0.43 | 0.038 | 0.574 | 0.505 |
| BCAA biosynthesis | BRANCHED-CHAIN-AA-SYN-PWY | Superpathway of branched chain amino acid biosynthesis | 0.80^a^ | 0.64^b^ | 0.71 | 0.003 | 0.234 | 0.959 |
| Ile biosynthesis | ILEUSYN-PWY | L-isoleucine biosynthesis I (from threonine) | 0.91^a^ | 0.72^b^ | 0.88 | 0.007 | 0.721 | 0.505 |
|  | PWY-3001 | Superpathway of L-isoleucine biosynthesis I | 0.67^a^ | 0.57^b^ | 0.64 | 0.005 | 0.279 | 0.721 |
|  | PWY-5101 | L-isoleucine biosynthesis II | 0.95^a^ | 0.75^b^ | 0.88 | 0.005 | 0.442 | 0.505 |
|  | PWY-5103 | L-isoleucine biosynthesis III | 0.75^a^ | 0.61^b^ | 0.64 | 0.007 | 0.195 | 0.959 |
|  | PWY-5104 | L-isoleucine biosynthesis IV | 0.40 | 0.43 | 0.49 | 0.328 | 0.878 | 0.798 |
| Val biosynthesis | VALSYN-PWY | L-valine biosynthesis | 0.91^a^ | 0.72^b^ | 0.88 | 0.007 | 0.721 | 0.505 |

*, Category and Pathway ID and description were derived from the MetaCyc database (https://metacyc.org/). I, initial raw vegetable group; P, pretreatment group; S, salt stock preparation group; BCAA, branched-chain amino acid. Different superscript letters in a row indicate a significant difference between the mean values (*P* < 0.05).
