# Supplementary material for: The effects of vegetable pickling conditions on the dynamics of microbiota and metabolites
Source: PeerJ. 2021 Apr 6;9:e11123. doi: 10.7717/peerj.11123 (PMC8034358; doi:10.7717/peerj.11123)
Supplement: Supplemental Information 6 — First row, Desulfococcus oleovorans; second row, Halanaerobium congolens. The alignments were obtained from Protein BLAST (NCBI, https://blast.ncbi.nlm.nih.gov/Blast.cgi. The amino acids shown in red are the critical residues for NADPH/NADH selectivity (Brinkmann-Chen, Cahn & Arnold, 2014). Reference Brinkmann-Chen S, Cahn JKB, Arnold FH. 2014. Uncovering rare NADH-preferring ketol-acid reductoisomerases. Metab Eng 26 :17–22. [file peerj-09-11123-s006.pdf]

```
Desulfococcus oleovorans  27   VLKNEVITVLGYGVQGPAQALNLKDNGFEVIIGQLEGDAYWEKAIADGFVPGKTLFPIEE   86
                               VL +  + V+GYG QG AQALN++D+G E II    GD+  E+A  DGF      + +++
Halanaerobium congolens   16   VLDDRTVAVIGYGNQGRAQALNMRDSGVENIIIGNRGDSSKEQAKKDGF----EAYSMDQ   71

D. oleovorans             87   AAKKGTIIKMLLSDAGQVAVW-PKVKKCLKKGDALYFSHGFGIVYKDQTGIVPPKNVDVI   145
                               AA K  I+  L+ D  Q  V+  K++  LK+GD L F+ G+ I Y     I PP+N+DVI
H. congolens              72   AANKADILFFLVPDEVQPQVYKEKIEPYLKEGDVLNFASGYNITY---NLITPPENIDVI   128

D. oleovorans             146  LVAPKGSGTNVRRNFKDGSGINSSYAVFQDATGRAEERTIALGIAIG---SGYLFPTTFE   202
                               ++AP+  GT VR  ++ G G  S  A+ Q+ +G+A++  +ALG AIG   SG +   +F
H. congolens              129  MIAPRMIGTMVRELYEVGDGAPSFLAINQNYSGKAKDIGLALGKAIGSTRSGIIEIDSFA   188

D. oleovorans             203  KEVFSDLTGERGVLMGCLAGTMEAQYNVLRKHGHSPSEAFNETVEELTQSL---IRLVAE   259
                                E  SDL  E+G L+  +   M A+Y +    G  P+ A  E    L++ L      +AE
H. congolens              189  LETKSDLLMEQG-LIPIILNAMIAKYELEIAEGMPPAGALLELY--LSRELGYIFEKMAE   245

D. oleovorans             260  NGMDWMFANCSTTAQRGALDWAPKFRDAVA   289
                                G+       S T+Q G +    + ++  A
H. congolens              246  QGIIGQMPLHSQTSQYGQISRIDELQEGEA   275
```
